# Transforming Growth Factor Beta is regulated by a Glucocorticoid-Dependent Mechanism in Denervation Mouse Bone

**DOI:** 10.1038/s41598-017-09793-y

**Published:** 2017-08-30

**Authors:** Ye Li, Ligang Jie, Austin Y. Tian, Shenrong Zhong, Mason Y. Tian, Yixiu Zhong, Yining Wang, Hongwei Li, Jinlong Li, Xiaoyan Sun, Hongyan Du

**Affiliations:** 1 School of Laboratory Medicine and Biotechnology, Southern Medical University, Guangzhou, China; 2 Department of Chinese Medicine, Guangzhou General Hospital of Guangzhou Command, PLA, Guangzhou, China; 3 Department of Neurology, University of Chicago, Chicago, IL USA; 4 College of Dentistry, University of New York, New York, NY USA

## Abstract

Bone growth and remodeling is inhibited by denervation in adults and children, resulting in alterations of linear growth and bone mass and increased risk for osteoporosis and pathologic fractures. Transforming growth factor beta (TGF-β) isoforms are a key group of growth factors that enhance bone formation. To explore the relation between denervation-induced reduction of bone formation and TGF-β gene expression, we measured mRNA levels of TGF-β in denervation mouse bone and found decreased mRNA levels of TGF-β1, TGF-β2 and TGF-β3. These changes were accompanied by diminishing weight loss, bone mineral density (BMD), trabecular thickness, trabecular separation and trabecular number of femur and lumbar, serum osteocalcin, total calcium, intact parathyroid hormone, and increased serum C telopeptide. Recombinant human TGF-β1 (rhTGF-β1) prevented denervation-induced reduction of BMD further supporting our hypothesis that denervation-induced reduction of bone formation is a result of inhibition of TGF-β gene expression. In addition, antiprogestins RU 38486 blunted the denervation-induced decrease in mRNA levels of TGF-β group, while dexamethasone (DEX) decreased TGF-β group mRNA levels in normal mice. Furthermore, the denervated-mice exhibited a threefold increase in plasma corticosterone. These results suggest that denervation-induced reduction of bone formation may be regulated by glucocorticoids via inhibition of TGF-β gene expression at least in part.

## Introduction

Denervation is usually caused by trauma, anesthesia, unloads after chronic severe diseases and side effects of medicine etc, and its metabolic consequences are of great clinical significance. One of the most significant metabolic consequences of denervation is impairing skeletal bone formation. Evidence from both clinical and experimental results demonstrates that bone metabolism is abnormal following denervation^1, 2^. Moreover, a cross-sectional study of the lumbar spine of children with denervation showed that bone mineral density (BMD) was significantly lower in physical disabled children than in age-related normal children^3, 4^. The consequences of BMD reduction include an increase in extrapolated annual fracture incidence, reduced peak bone mass, adult-onset osteoporosis, and delay in growth velocity.

The most promising clinical agent for preventing the reduction of bone formation is recombinant human growth hormone, which can stimulate bone formation via production of insulin-like growth factor and its associated binding protein, which correlates with BMD in children^5^. However, little attention has been directed toward defining local and systemic regulators of bone metabolism after denervation including growth and differentiation factors, cytokines, hormones, and extracellular matrix components. Among these factors, the TGF-β/activin/BMP cytokine family is thought to play a major role in bone metabolism after denervation.

TGF-β, a family of multifunctional protein deposited in bone matrix by osteoblasts and released during osteoclastic resorption, may be an important regulator of osteoblast and osteoclast activity during bone growth and remodeling^6–9^. *In vivo*, TGF-β has been implicated as a regulator of endochondral ossification during both skeletal formation^10^ and fracture healing^11^. TGF-β is expressed at all stages during endochondral bone formation, and exogenous TGF-β has dramatic effects on gene expression and differentiation of cartilage and bone cells during bone repair^11, 12^. Furthermore, *in vivo* administration of TGF-β induces rapid closure of skull defects^13^, callus formation in normal bone, and increased bone formation and strength during rat tibial fracture repair^11, 12, 14, 15^. While these findings demonstrate TGF-β regulation of osteoblast and osteoclast function, the role of endogenous TGF-β in bone formation and remodeling remains unclear.

Excessive endogenous glucocorticoids (GCs) production as an underlying cause of reduced bone formation has been recognized for more than 60 years^16^, but the precise cellular and molecular basis of these changes has remained elusive. Plasma levels of GCs are elevated both in patients^17^ and experimental animals after denervation^18^. Administration of dexamethasone (DEX) in human or animal resulted in increased reduction of bone formation^19, 20^. Further evidence for a role of GCs in reduction of bone formation was elicited in experiments in which treatment of mouse and human osteoblast cell lines with the glucocorticoid receptor (GR) antagonist RU 38486 significantly blunted GC-decreased numbers of functional osteoblasts^21^. Therefore, we hypothesize that denervation-reduced bone formation is a result of inhibition of TGF-β gene expression which may be regulated by GCs.

To test this hypothesis, the levels of BMD and TGF-β mRNA in bone from denervation mice treated with RU38486 were determined. In other experiments, the same measurements were performed after treatment of normal mice with DEX. Treatment with RU 38486 blunted the denervation-induced decrease in BMD and TGF-β mRNA levels, while treatment of normal mice with DEX reduced BMD and TGF-β mRNA levels. In addition, the denervation mice exhibited a twofold decrease in plasma TGF-β during bone loss period, and rhTGF-β1 prevented denervation-induced BMD. These observations support the hypothesis that GCs at least in part regulate denervation-induced reduction of bone formation by inhibiting TGF-β gene expression in skeletal tissue.

## Results

### Denervation induced weight loss in mice

**Figure 1:**
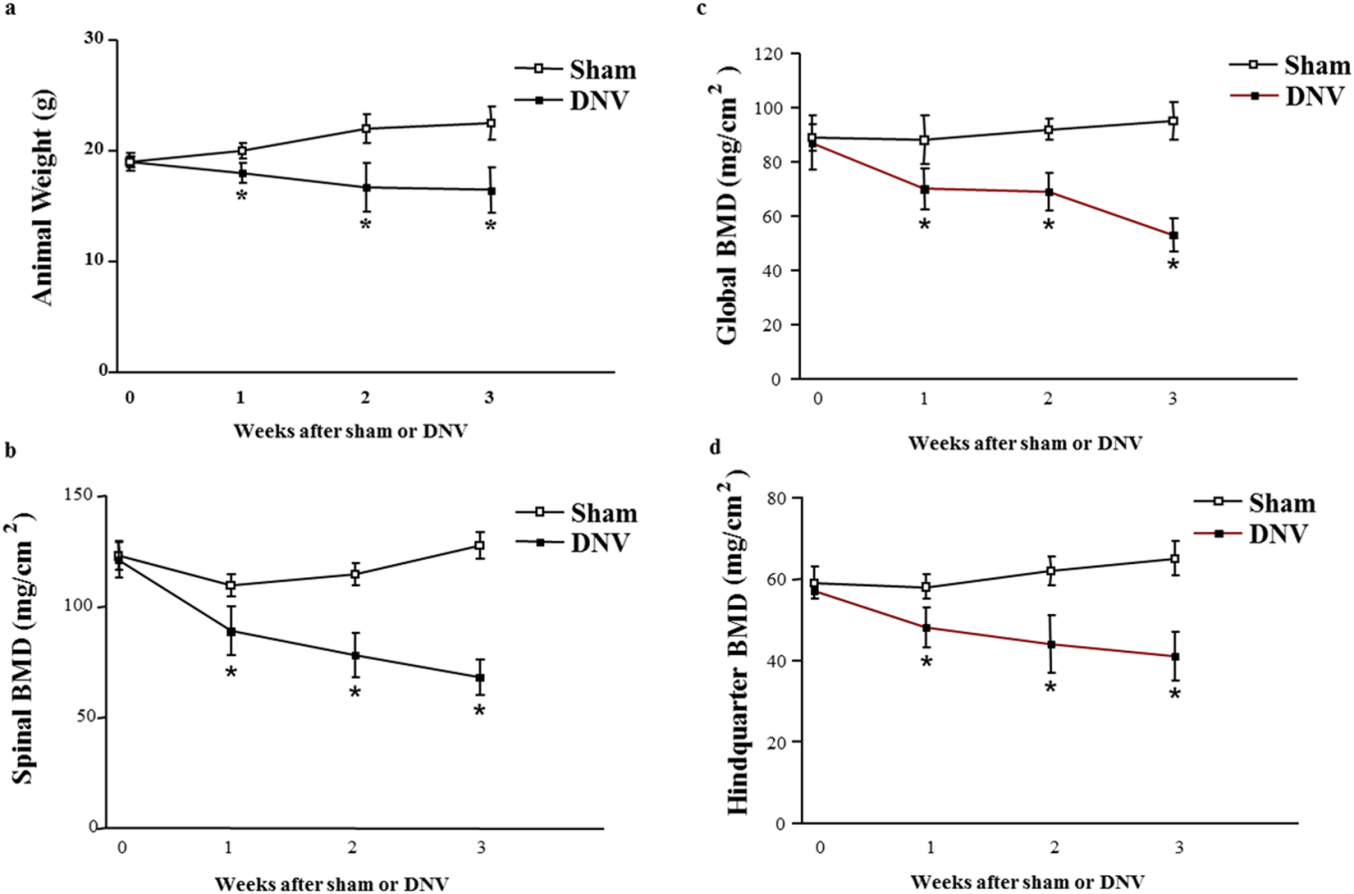
Effect of denervation on total body weight. (**a**) Body weight and BMD levels in spinal (**b**), global (**c**), and hindquarter (**d**) bones in sham-operated and sciatic nerve crushed mice. Data expressed as means ± SEM (*n* = 7) with *p < 0.05, sham *vs* denervation at the same time point by Student’s *t*-test. The time point 0 h represents normal, unoperated rats.

The total body weight gain was measured to assess denervation-induced bone loss and muscle cachexia. The results showed that sciatic nerve crush resulted in a significant decrease in total body weight gain in mice in comparison with sham-operated animals, consistent with our recent previous results in the denervated-mice. Significant weight loss occurred at 1 week after sciatic nerve crush and was approximately 27% less than in sham-operated animals at 3 weeks (Fig. 1a).

### Denervation reduced bone formation in mice

**Figure 2:**
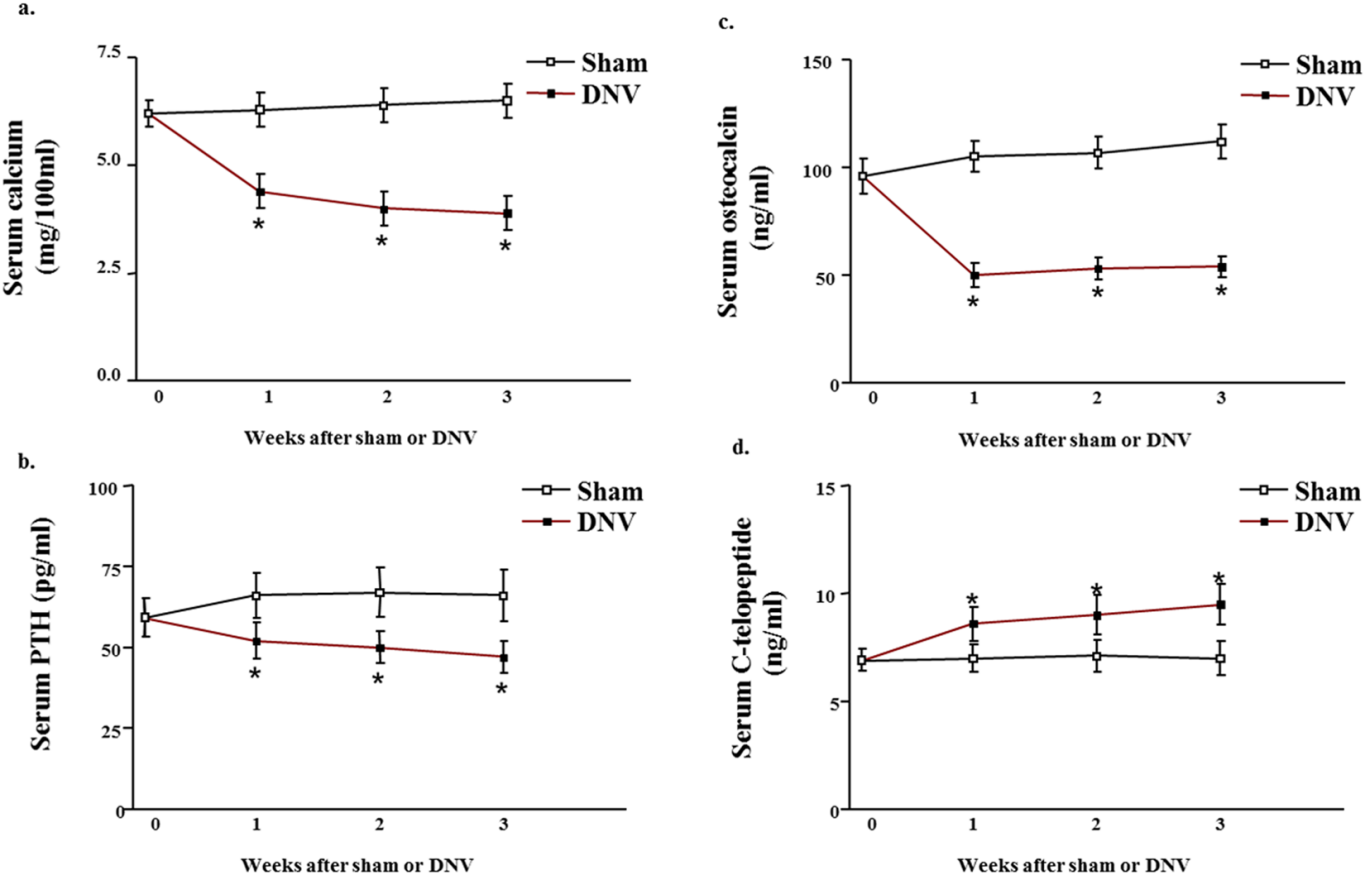
Effect of denervation on serum calcium. (**a**) PTH (**b**), osteocalcin (**c**), and C-telopeptide (**d**) levels in sham-operated and sciatic nerve crushed mice. Data expressed as means ± SEM (*n* = 7) with *p < 0.05, sham *vs* denervation at the same time point by Student’s *t*-test. The time point 0 h represents normal, unoperated rats.

In sciatic nerve crushed mice, spinal and global BMD levels were significantly lower than those found in the sham-operated mice at 1 week (Fig. 1b and c). The largest decrease was evident at 3 weeks, reflecting severe osteopenia. Sciatic nerve crush-induced loss of BMD was less evident in the hindquarters (Fig. 1d) than at the spinal and global sites, demonstrating the expected propensity for the axial skeleton. Additionally, the trabecular thickness, trabecular separation and trabecular number of femur and lumbar in sciatic nerve crushed mice markedly decreased comparing to in sham-operated mice (Fig. S1a,b). Meanwhile, the average cortical thickness of cortical bone of femur also decreased in sciatic nerve crushed mice (Fig. S1c). Serum total calcium and intact PTH concentrations in the sciatic nerve crushed-mice were reduced at 1 week compared to sham-operated mice and remained low throughout the denervation period (Fig. 2a and b). These results are consistent with those in denervation patients^22, 23^, suggesting that either bone deposition or bone resorption, or a combination of both, is altered in denervated-animals with decreased bone content.

To test the effect of denervation on bone formation as well as resorption parameters *in vivo*, we measured serum osteocalcin, a marker of osteoblast activity, and serum C-telopeptide, a marker of osteoclast activity. Levels of serum osteocalcin were significantly decreased and those of serum C telopeptide were increased in sciatic nerve crushed-mice compared with sham-operated mice. The changes were obvious at 1week after sciatic nerve crush and lasted throughout the denervation period (Fig. 2c and d), suggesting that denervation reduces bone formation but increases bone resorption.

### Denervation decreased TGF-β mRNA in mice bone

**Figure 3:**
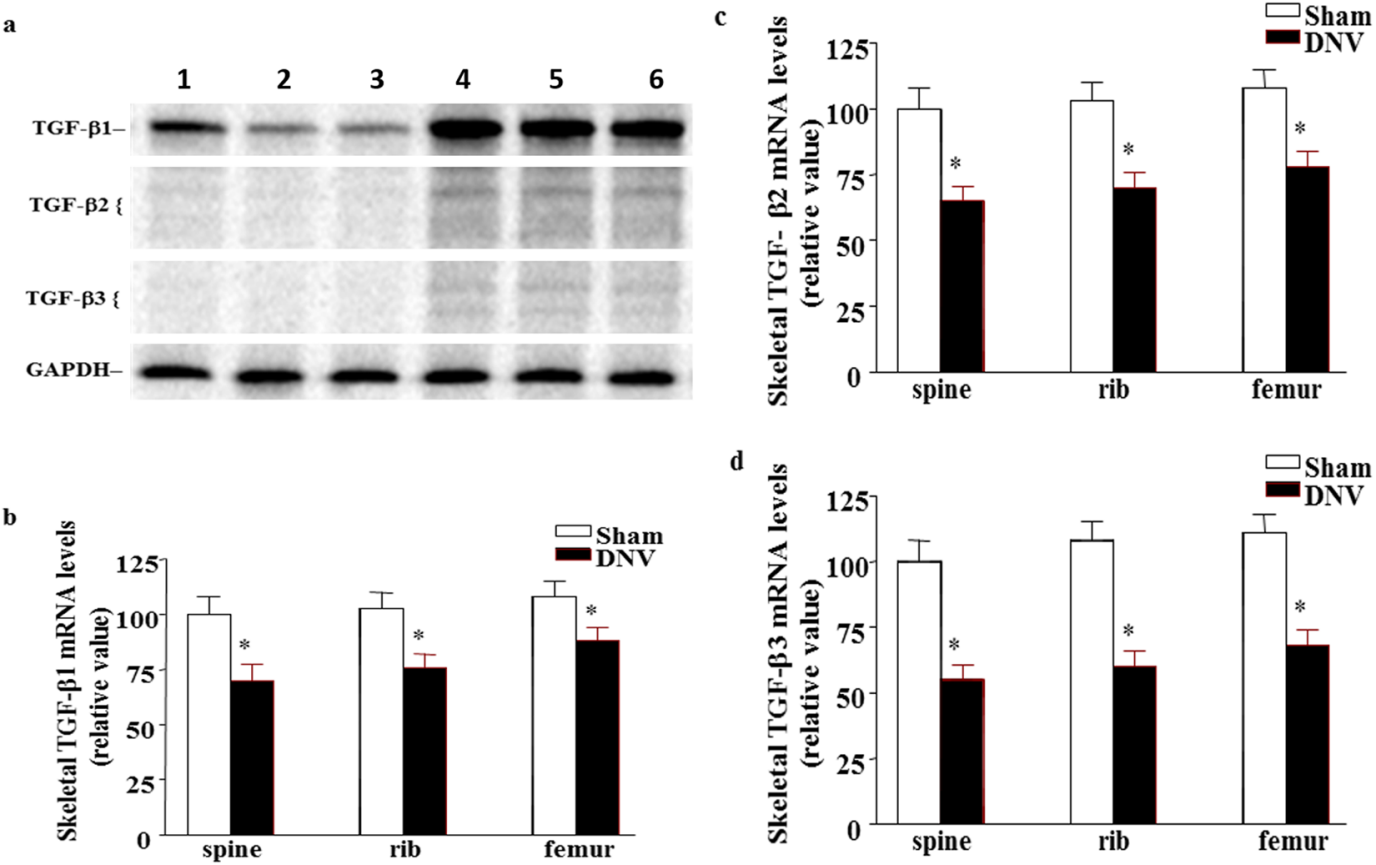
Quantitative analysis of mRNA expression for multiple TGF-β genes. (**a**) Representative autoradiographic images of the RPA products as resolved on a 6% PAGE sequencing gel. Lane 1, 2 and 3 present sciatic nerve crushed and lane 4, 5 and 6 present sham-operated at 3 weeks in spine, rib and femur respectively. (**b**–**d**) The graphic analysis of the relative mRNA levels. Band densities were determined from several different autoradiographic exposures and taken from the linear range of the film exposure. Band densities were normalized to the ratio of the internal standard GAPDH and expressed as a relative value. Results are means ± SEM (*n* = 7 in each group) with *p < 0.05, sham *vs* denervation mice at the same bone site by Student’s *t*-test.

TGF-β1, TGF-β2 and TGF-β3 gene expression in spine, rib and femur of mice at 3 weeks after sciatic nerve crush was measured by using RPA (Fig. 3). TGF-β1 was the most expressed gene in control (sham-operated) mice bones. The reduction of TGF-β1, TGF-β2 and TGF-β3 mRNA levels all occurred in spine, rib and femur of sciatic nerve crushed-mice compared with those in sham-operated mice. Among those changes, the largest decrease occurred in TGF-β3 mRNA levels 3 weeks after denervation.

### Effect of GCs on denervation-induced reduction of bone formation

**Figure 4:**
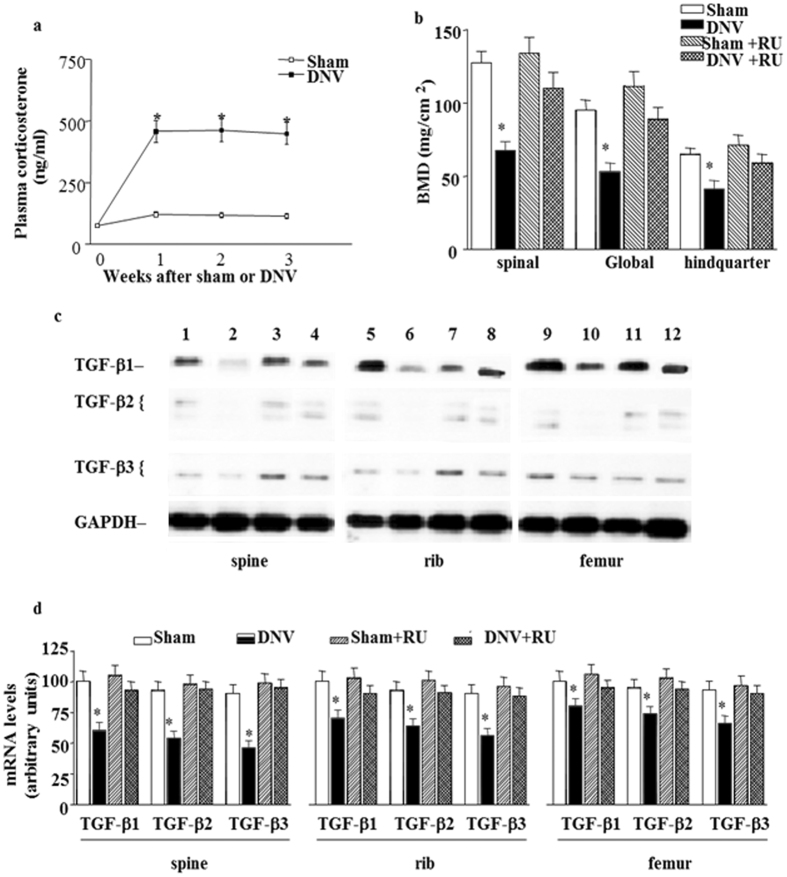
Effect of GCs on denervation-induced reduction of bone formation. (**a**) Effect of denervation on plasma corticosterone levels in sham-operated and sciatic nerve crushed mice. Data expressed as means ± SEM (*n* = 7) with *p < 0.01, sham *vs* denervation at the same time point by Student’s *t*-test. The time point 0 h represents normal, unoperated rats. (**b**) RU 38486 (RU) prevented denervation decreased BMD levels in spinal, global and hindquarter bone at 3 weeks. Data expressed as means ± SEM (*n* = 7) with *p < 0.05 among all groups within each bone site by *ANOVA*. (**c**) Representative autoradiographic images of the RPA products as resolved on a 6% PAGE sequencing gel. Lane 1, 5 and 9 present sham; lane 2, 6 and 10 present denervation; lane 3, 7 and 11 present sham with RU 38486 treatment; and lane 4, 8 and 12 present denervation with RU 38486 treatment at 3 weeks in spine, rib and femur respectively. (**d**) Densitometric analysis of TGF-β multiple gene expression levels in spine, rib and femur respectively corrected for GAPDH. Data are means ± SEM (*n* = 7) with *p < 0.05 among all groups by *ANOVA*.

To elucidate the potential role of GCs on denervation-induced reduction of bone formation, plasma corticosterone was examined. The plasma corticosterone level was dramatically increased by 5-fold at 1 week and throughout the denervation period (Fig. 4a). To further investigate the role of GCs on denervation-induced reduction of bone formation, BMD levels were measured in mice treated with RU 38486. The results showed that RU 38486 prevented denervation-induced reduction of bone formation (Fig. 4b). We next examined the effect of RU 38486 on the denervation-induced decrease in gene expression of TGF-β. Again, RU 38486 blunted the denervation-induced decrease in TGF-β1, TGF-β2 and TGF-β3 mRNA levels in spine, rib and femur sites (Fig. 4c and d).

**Figure 5:**
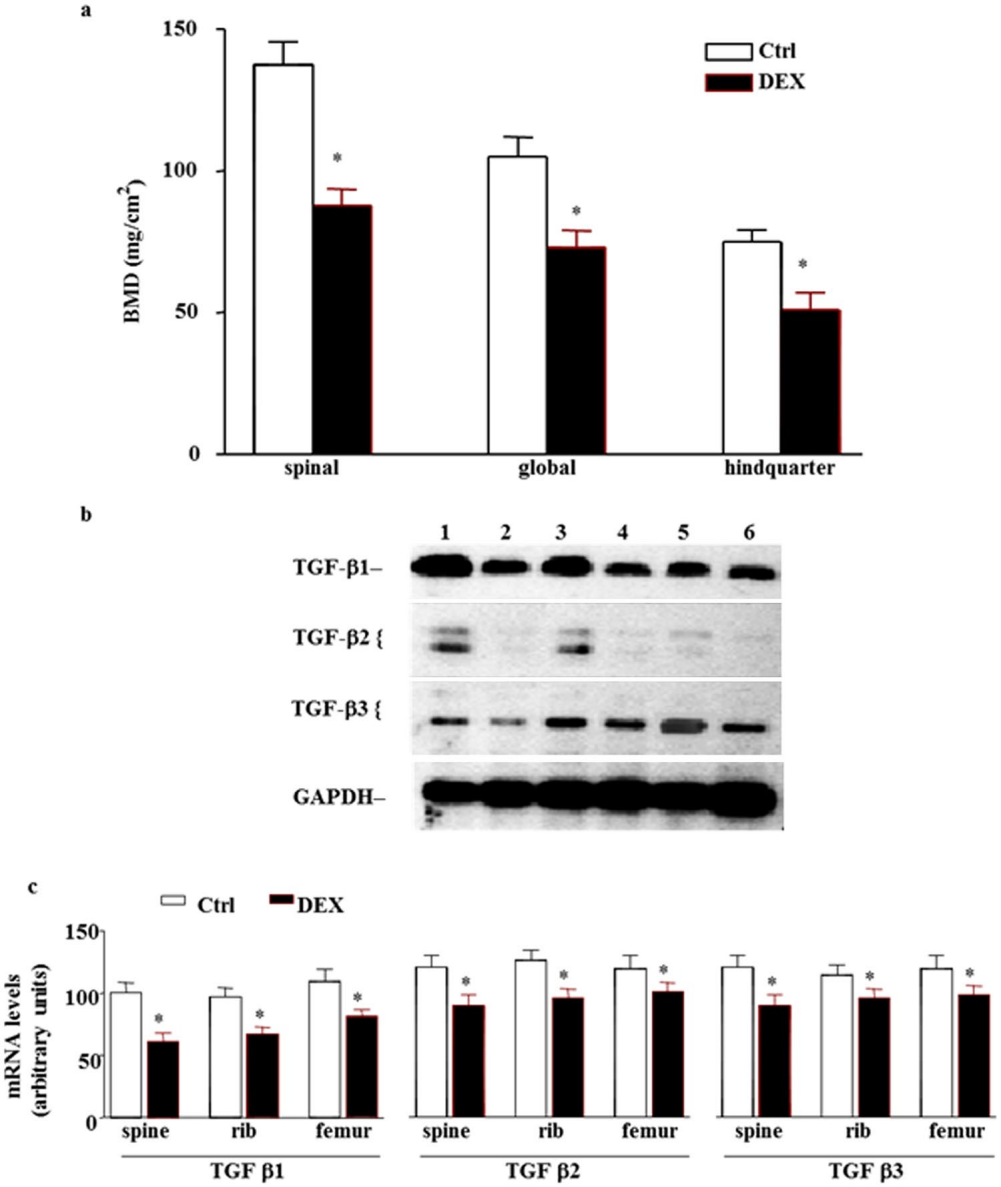
Effect of GCs on bone formation. (**a**) DEX decreased BMD levels in spinal, global and hindquarter bone in normal mice at 3 weeks. Data expressed as means ± SEM (*n* = 7) with *p < 0.05 *vs* control (Ctrl) by Student’s *t*-test. (**b**) Representative autoradiographic images of the RPA products as resolved on a 6% PAGE sequencing gel. Lane 1, 3 and 5 present Ctrl group, and lane 2, 4 and 6 present DEX-treated group in spine, rib and femur respectively. (**c**) Densitometric analysis of TGF-β1, TGF-β2 and TGF-β3 mRNA levels, respectively, corrected for GAPDH. Data are means ± SEM (*n* = 7) with *p < 0.05 *vs* Ctrl by Student’s *t*-test.

**Figure 6:**
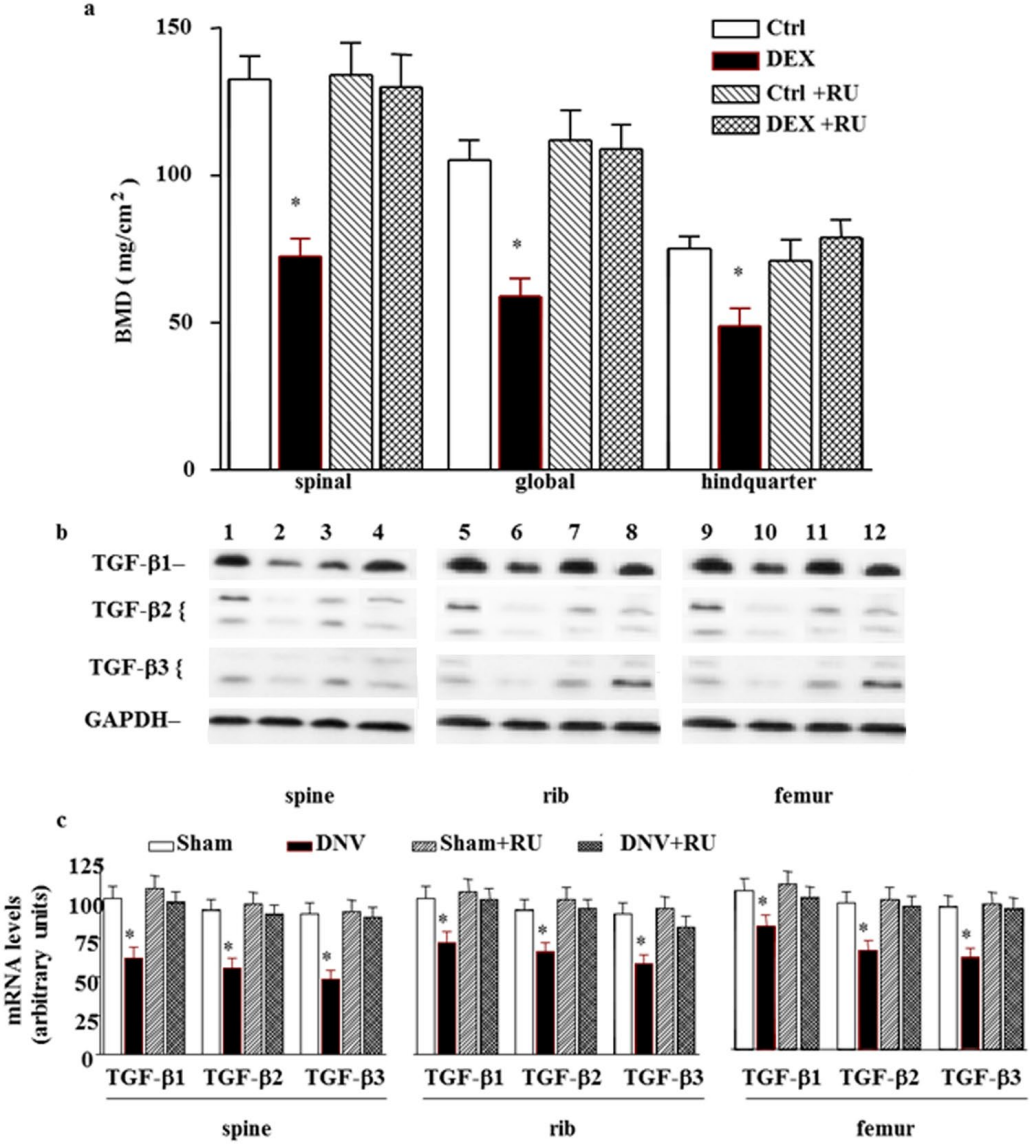
Effect of RU 38486 on DEX-induced reduction of bone formation. (**a**) RU 38486 (RU) blocked DEX decreased BMD levels in spinal, global and hindquarter bone in normal mice at 3 weeks. Data expressed as means ± SEM (*n* = 7) with *p < 0.05 among all groups within each bone site by *ANOVA*. (**b**) Representative autoradiographic images of the RPA products as resolved on a 6% PAGE sequencing gel. Lane 1, 5 and 9 present Ctrl; lane 2, 6 and 10 present DEX; lane 3, 7 and 11 present Ctrl with RU 38486 treatment; and lane 4, 8 and 12 present DEX with RU38486 treatment at 3 weeks in spine, rib and femur respectively. (**c**) Densitometric analysis of TGF-β multiple gene expression levels in spine, rib and femur respectively corrected for GAPDH. Data are means ± SEM (*n* = 7) with *p < 0.05 among all groups by *ANOVA*.

To further test the role of GCs in the regulation of denervation-induced reduction of bone formation, normal mice were treated with DEX. Injection of DEX resulted in a decrease of BMD at 3 weeks (Fig. 5a). We next examined whether the DEX-induced reduction of bone formation was associated with decreased TGF-β mRNA levels. Spine, rib and femur levels of TGF-β1, TGF-β2 and TGF-β3 mRNA were decreased after treatment with DEX (Fig. 5b and c). Finally, we tested the specificity of RU 38486 by administering the drug to mice treated with DEX. RU 38486 pretreatment completely abolished the DEX-decreased TGF-β1, TGF-β2 and TGF-β3 mRNA levels (Fig. 6b and c) while blunted DEX-resulted in a decrease of BMD at 3 weeks (Fig. 6a), suggesting that the effect of GCs on the gene expression of TGF-β is mediated by a GR. Meanwhile, histomorphometric data of for alkaline phosphatase (ALP) activity decreased and tartrate-resistant acid phosphatase (TRAP) activity increased in sciatic nerve crushed mice comparing to in sham-operated mice (Fig. S2a,b), which indicated that TGF-beta decreasing because of denervation was involved in both of a reduction in osteoblast function and increase in osteoclast at same time.

### Effect of rhTGF-β1 on denervation-induced reduction of bone formation

**Figure 7:**
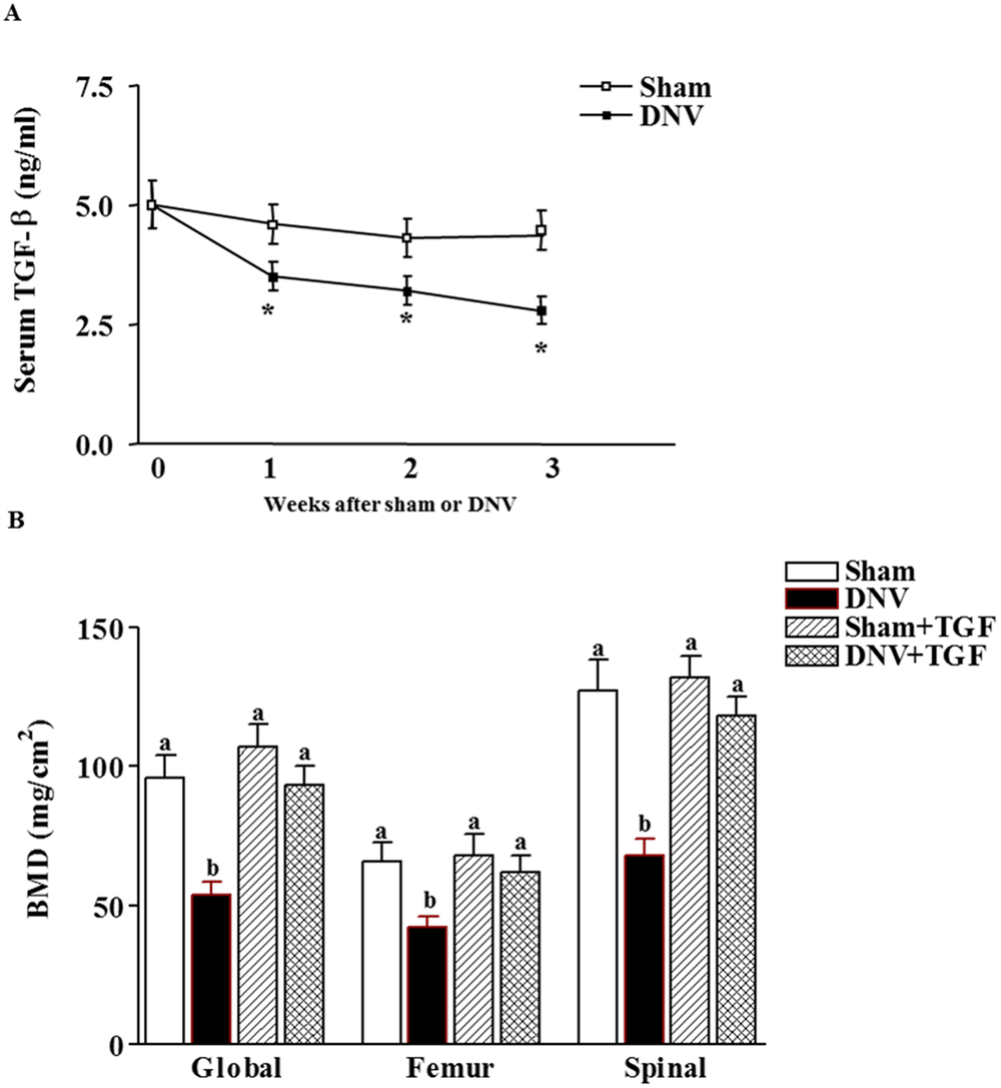
Effect of TGF-β on denervation-induced reduction of bone formation. (**a**) Effect of denervation on serum levels in Sham-operated and sciatic nerve crushed mice. Data expressed as means ± SEM (*n* = 7) with *p < 0.05, sham *vs* denervation at the same time point by Student’s *t*-test. The time point 0 h represents normal, unoperated rats. (**b**) rhTGF-β1 prevented denervation decreased BMD levels in spinal, global and hindquarter bone in mice at 3 weeks. Data expressed as means ± SEM (*n* = 7) with *p < 0.05 among all groups within each bone site by *ANOVA*.

To elucidate the potential role of rhTGF-β1 on denervation-induced reduction of bone formation, plasma TGF-β1 was examined. The plasma TGF-β1 level was significantly decreased by 27% at 1 week and throughout the denervation period (Fig. 7a). To further investigate the role of TGF-β on denervation-induced reduction of bone formation, BMD levels were measured in mice treated with rhTGF-β1. The results showed that rhTGF-β1 prevented denervation-induced reduction of BMD at 3 weeks (Fig. 7b), which is consistent with our recent study in which rhTGF-β1 prevented denervation-induced reduction of endochondral formation. These results suggest that denervation-induced reduction of bone formation may be regulated by inhibition of TGF-β.

## Discussion

This study demonstrates that: (1) denervation reduces bone formation and gene expression of TGF-β in skeletal tissue; (2) this effect is due at least in part to GCs; and (3) denervation-induced reduction of bone formation can be attributed to inhibition of TGF-β gene expression by GCs.

The C57BL/6J mouse strain chosen for these studies is the base for several transgenic mouse models used to characterize longitudinal changes in BMD with age and after denervation. As demonstrated in the present study, the C57BL/6J strain provides a valid mouse model for the study of denervation induced skeletal changes and catabolic alterations, although this strain has the lowest BMD among several different strains. A catabolic response to denervation was detected within 1 week at most skeletal sites. Denervation significantly decreased BMD at all sites, similar to the effects observed in humans and rats^3, 4, 23^, indicating that denervation-induced reduction of bone formation is a systemic reaction.

The pathogenesis of acute loss of bone mass after denervation is unclear, but our results provide further evidence of decreased serum osteocalcin in bone formation, increased serum type I collagen telopeptide in bone resorption, decreased BMD in bone loss, and decreased gene expression of TGF-β after denervation. This disorder of bone homeostasis may increase the susceptibility of denervated children or adults to limb and axial fractures long after nerve crush, as previously reported in humans and rat models with comparable denervation^2–4^. In addition, denervation-induced weight loss has been demonstrated in previous reports from this and other laboratories in which GCs were shown to play an important role in the catabolic response to denervation and other conditions including sepsis, burn-injury, cancer and starvation^24–26^. These results are supported by findings of increased GCs in these conditions and the prevention of muscle proteolysis by adrenalectomy or treatment with RU 38486^24–26^.

The aim of the current study was to provide quantitative characterization of the expression patterns for the TGF-β group of growth factors at 3 weeks in a murine denervation model. RPA provides accurate and sensitive quantification of mRNA expression^27^. However, a number of limitations must be considered when interpreting these results. First, the whole spine, rib or femur is a heterogeneous mixture of various cells and tissues, and RPA data does not address the spatial patterns of gene expression among these various cell types. Second, mRNA expression may be down-regulated in cells pre-existing in the tissue, or certain types of cells expressing a gene could be recruited during the recovery process, thus making it difficult to ascertain the origin of cells expressing a specific gene. Finally, there may be a strong reduction of a gene’s activity in a small number of cells or a moderate induction in a large number of cells. Nevertheless, the data reported here provides some insight into 3 weeks during which the TGF-β group may act as a number of factors for bone formation after denervation.

In this study we also examined the expression of the TGF-β group in sciatic nerve crushed mice. The role of TGF-β in chondrogenesis and osteogenesis has been investigated extensively, and the results demonstrate that these growth factors are produced by a variety of cells including osteocytes, osteoblasts, osteoclasts, and chondrocytes. Subperiosteal implantation of TGF-β in mice initiates endochondral ossification^28^, and several investigators have documented the expression of TGF-β in fracture healing^29, 30^. However, the role of TGF-β on bone formation after denervation remains unclear. Therefore, we utilized multiprobe RPA to compare the relative expression of each of the isoforms and found that TGF-β1 has a high basal level of expression in normal bone, while TGF-β2 and TGF-β3 require induction. TGF-β1 expression occurs low at 3 weeks after denervation. TGF-β2 and TGF-β3 expressions dropped lower than TGF-β1 after denervation at 3 weeks, consistent with previous reports that TGF-β2 and TGF-β3 were more active than TGF-β1 during new bone formation and chondrogenesis^28, 31^. In addition, rhTGF-β1 treatment prevented denervation-reduced BMD, which is consistent with our recent report that denervation-induced reduction of endochondral formation was reversed with rhTGF-β1 treatment. These observations support our hypothesis that denervation-induced reduction of bone formation may be due to inhibition of the TGF-β gene regulation pathway.

A variety of factors may affect acute bone loss after denervation, including immobilization^32–34^, aluminum loading^35, 36^, cytokines such as IL-1β, IL-6 and TNF-α^2, 37, 38^,and GCs^34^. Among these factors, GCs play an important role in denervation-induced reduction of bone formation by impairing osteoblast recruitment and function^39^, increasing bone resorption by reducing intestinal calcium absorption and thus producing secondary hyperparathyroidism^40^, consistent with our current results (Fig. 2), and by stimulating production of bone-resorbing cytokines such as interleukin-6^41^. Our present study shows that GCs inhibit TGF-β gene expression in skeletal tissue and that reduced bone formation may be due to inhibition of the TGF-β signal pathway by GCs.

In this study, treatment of rats with RU 38486 blocked denervation-induced reduction of bone formation and TGF-β mRNA levels. The data strongly support the hypothesis that GCs are involved in the regulation of bone formation after denervation and are in line with previous reports suggesting that GCs may decrease bone formation and result in osteoporosis^42^. Further support for this concept was demonstrated in the current study in which treatment of normal mice with DEX resulted in decreased BMD at the spine, global and hindquarter site, as well as decreased skeletal levels of TGF-β mRNA spine, rib and femur.

The mechanism of down-regulated TGF-β gene expression in denervated skeletal tissue is not fully understood, but results from the experiments in which denervation was induced in mice treated with RU 38486 strongly suggest that elevated GC levels play a significant role. GCs also play an important role in poor nutrition and low body weight, disorders of the neuroendocrine axis, immobilization, and other chronic illness^43^. The present results are important because they suggest that denervation or DEX treatment decreases bone formation, similar to previous reports in which GC treatment of both humans^20^ and experimental animals^44, 45^ altered growth and bone turnover. Our results are similar to those in a previous report^46^ suggesting that bone formation is particularly sensitive to the effect of GCs.

In this study, skeletal TGF-β mRNA levels were decreased in normal (non-denervated) mice after the administration of DEX. Two recent reports showed that administration of DEX decreased human lung fibroblast levels of TGF-β1 and TGF-β2 mRNA^47^ and inhibited TGF-β mediated promoter in mouse fibrosarcoma L929 cells^48^. Those studies support the use of DEX in the current study to induce a decrease in TGF-β mRNA levels. We studied the effect of GCs *in vivo* in normal mice and confirmed that GCs play an important role in mediating TGF-β mRNA expression.

It should be noted that although RU 38486 is a potent GR antagonist, the drug is not completely specific. Thus, in addition to blocking the GR, it also blocks the progesterone receptor^49^. Previous studies showing that progesterone prevents ovariectomy-induced bone loss, similar to the effects of RU 38486^50^, and inhibits the stimulation of neonatal mouse calvarial bone resorption by DEX in a competitive manner^51^ suggests an agonist-like effect of progesterone on bone formation. In addition, it has been reported that plasma progesterone levels are reduced after denervation^52^. Therefore, the effect of RU 38486 noted in the current study could exclude from blockade of progesterone receptors. In addition, recent studies suggesting that RU 38486 may act as an antioxidant^2^ may assist interpretation of the present results since oxygen free radicals are generated after denervation^53^ and there is a positive correlation between antioxidant intake and bone mineral density^54, 55^. However, the results in the present study of decreased bone formation after treatment of mice with DEX and inhibition of this effect by RU 38486 support the interpretation that the effects of RU 38486 in denervation mice were caused, at least in part, by blockade of the GR.

The mechanism by which GCs inhibit the TGF-β signal pathway is not clear from the present study. It is well known that GCs activate or inhibit gene transcription by binding to cytosolic GRs and forming a complex which translocates to the nucleus. In the nucleus, this complex acts as a transcriptional activator or inhibitor by binding to a GC response element (GRE) in the promoter of a target gene. When we analyzed the promoter regions of TGF-β genes using the MatInspector V2.2 program^56^, our results showed that the promoters of the human (GenBank Accession # J04431), rat (GenBank Accession # AF239170 and # AF249327) and mouse (GenBank Accession # M57902) TGF-β1 gene have binding sites for consensus GRE (5′-AGAACA). These observations support the hypothesis that GCs may be involved in the regulation of TGF-β genes inhibited in skeletal tissue after denervation by a GR pathway.

The results in this study are important from a clinical standpoint because reduction of bone formation is a significant metabolic response in denervated patients. Our data showing that acute osteopenia was maintained indicates that mice did not completely recover from the acute insult and that those entering the period of peak bone mass accumulation may develop lifelong osteoporosis. Thus, the denervation-induced bone loss may be a forerunner of early-onset osteoporosis in later life, but a longitudinal study is needed to test this hypothesis. Although most previous reports of increased reduction of bone formation after denervation were based on human and animal studies, we found evidence of decreased TGF-β gene expression in skeletal tissue from denervation mice and that rhTGF-β1 treatment prevented denervation-induced reduction of bone formation, suggesting that the TGF-β signal pathway is important for bone catabolism in humans as well. However, the precise role of the TGF-β superfamily in bone formation after denervation or other injury is unknown. Further studies will include a molecular biological study of TGF-β in bone formation and possible interventions to prevent the acute bone loss following denervation. Although GCs are associated with reduced bone formation, little is known about their molecular mechanism in skeletal metabolism. A better understanding of the regulation of GC-dependent bone catabolism via the TGF-β signal pathway may lead to future metabolic management of patients with denervation and perhaps other catabolic conditions as well.

## Experimental Work

### Denervation Animal Model

All the animals’ experiments were approved by Animal Care and Ethics Committee of Guangzhou center for disease control and prevention. Denervation was created as previously described^57^ with minor modification by bilateral sciatic nerve crush. Briefly, 5-week old male C57BL/6 mice (Harlan Company, Indianapolis, IN) weighing 16–18 g were acclimated for 1 week at an ambient temperature of 25 °C with a 12 h light/dark cycle and fed rodent chow and water ad libitum. The 6-week-old mice were divided into sham and bilateral sciatic nerve crush groups (n = 7).

Mice were weighed and then killed quickly by cervical dislocation. Approximately 0.2 ml of trunk blood was collected over intervals of 1, 2, and 3 weeks into centrifuge tubes containing 20 μl of 0.3 M EDTA for immunoassay. Collected blood was immediately centrifuged by using a Sorvall RC-3 centrifuge at 4 °C for 15 min at 3,000 g. The supernatant was stored at −70 °C before use. The spine, rib and femur were harvested at 3 weeks after sham-operation or sciatic nerve crush and immediately frozen in liquid nitrogen. In order to diminish the bone site specificity for denervation, the spine, rib and femur were studied because previous reports demonstrated that bone formation and osteoblast number are reduced both in rat limb and mouse femur 3 weeks after the denervation. The bones were stored at −70 °C until analysis of TGF-β.

### Treatment of mice with DEX

Six-week old male C57BL/6 normal mice were treated with DEX dissolved in 1 mg/ml of PBS (stock solution, pH 7.4) and administered subcutaneously at a dose of 1.0 mg/kg/day. Control mice received a corresponding volume of PBS sq. The mice were housed and fed the same as the sciatic nerve crushed group, and metabolic studies were performed after 3 weeks to effect conditions similar to those in the sciatic nerve crushed mice. The doses of DEX were based on previous studies in which decreased bone formation resulted^58^.

### Treatment of mice with RU38486

Mice were treated by gavage with 10 mg/kg/day of RU 38486 (Research Biochemicals International, Natick, MA) or a corresponding volume of vehicle 2 h before sham-operated or denervation or the *sq* injection of DEX as described in detail previously^8, 59^. Metabolic studies were performed 3 weeks after sciatic nerve crush. The dose of RU 38486 is consistent with the dosage used to block DEX-induced metabolic changes in a previous study^60^.

### Treatment of mice with rhTGF-β1

Mice were administered with 100 μg/kg/day of rhTGF-β1 by *sq* injection or a corresponding volume of vehicle 2 h before sham or sciatic nerve crush as described in detail previously. Metabolic studies were performed 3 weeks after sciatic nerve crush. The bioactivity of rhTGF-β1 was confirmed using the mink lung inhibition assay as described previously^61^.

Unless indicated otherwise, all chemicals were purchased from Sigma Chemical Co. (St. Louis, MO). All mice were cared for in accordance with the National Research Council’s Guide for the Care and Use of Laboratory Animals. The experiments were conducted at University of Chicago, laboratory animal facility, in barrier housing within AAALAC-accredited animal quarters under protocols reviewed and approved by the Institutional Animal Care and Use Committee.

## RNase protection assay (RPA)

### Total RNA preparation

Mouse spine, rib and femurwere removed and the soft tissues trimmed away. The remaining skeletal tissue was frozen in liquid nitrogen and stored at −80 °C until used for RNA isolation. The specimens were powdered in liquid nitrogen using a mortar and pestle. Total RNA was extracted from the bone powder by the procedure of Chomczynski and Sacchi^62^ with Trizol (Gibco BRL, Gaithersburg, MD). RNA was treated with RQ1 RNase-free DNase (Promega Biotech, Madison, WI) for 30 min at 37 °C to remove contaminating genomic DNA. RNA was then monitored by visualization of ribosomal RNA with ethidium bromide after denaturing RNA gel electrophoresis.

### Probe preparation

Linearized plasmids containing TGF-β1, TGF-β2 and TGF-β3, and glyceraldehyde-3-phosphate dehydrogenase (GAPDH, an internal control) gene segments used for RPA were purchased from PharMingen Corp. (San Diego, CA). Single-stranded [^32^P]-labeled complimentary RNA (cRNA) probes were generated by *in vitro* transcription (PharMingen Corp.). Briefly, linearized plasmids containing each of the selected genes for analysis were transcribed with the use of [^32^P] uridine triphosphate (UTP; NEN life Science Products, Inc., Boston, MA) and T7 RNA polymerase. The labeling efficiency for the cRNA products was determined by scintillation counting and adjusted to a concentration of 3 × 10^5^ cpm/μl of probe for each RPA. The size of the protected fragments was determined by comparison to non-RNase-treated samples that were run as a ladder.

### RPA and data processing

RPA was performed using a RiboQuant ribonuclease protection kit (PharMingen Corp.) following the manufacturer’s instructions. Scanning and quantitation were performed in a phosphor imager using the Image Quant Program (Molecular Dynamics, Sunnyvale, CA) and the TGF-β1, TGF-β2 and TGF-β3 mRNA abundance was calculated as the ratio between TGF-β1, TGF-β2, TGF-β3 and GAPDH mRNA respectively and expressed as arbitrary units.

### Measure of bone mineral density

Dual energy X-ray absorptiometry (DEXA) was used to determine global (whole body minus the head), spinal and hindquarter BMD in live mice using a PIXImus mouse densitometer (Lunar, Maddison, WI) with small-animal software (version 1.44) according to previous reports^63^. Four experimental groups of mice were placed on a plastic attenuator under slight ether anesthesia at 1 to 3 weeks after sciatic nerve crush, RU 38486 or DEX treatment. The result of BMD was expressed as bone mineral content (BMC/area, mg/cm^2^).

### Serum biochemical measurements

Serum calcium was analyzed colorimetrically on an Ektachrome 7000 analyzer (Eastman Kodak Co., Rochester, NY)^64^. Serum osteocalcin was measured with RIA by using a goat anti-murine osteocalcin and murine osteocalcin as tracer and standard (Biomedical Technologies, Stoughton, MA) as described previously^65^. Serum intact PTH was measured using a commercial kit (Nichols Institute) according to the manufacturer’s instructions^63, 66^. Type I collagen telopeptide (bone resorption marker) was measured using a Rat ICTP RIA Double Antibody Kit (DiaSorin, Inc. Stillwater, MN) according to the manufacturer’s instructions. The sensitivity of this assay is 0.5 μg/liter, the mean recovery is 108.5%, and the intra- and interassay coefficients of variation are less than 11%^67, 68^. The corticosterone concentrations were measured using commercially available radioimmunoassay kits (ICN Biochemicals Inc., Costa Mesa, CA) as previously described^24^. The concentration of plasma TGF-β1 was measured with enzyme-linked immunosorbent assay kits (TGF-β1 kit, Genzyme, Cambridge, MA). Among the isoforms, TGF-β1 has been conventionally measured to reflect plasma TGF-β concentration^69, 70^.

### Statistics

Results are given as means ± SEM. Student’s t-test or ANOVA was used for statistical analysis as appropriate.

## Electronic supplementary material

supplementary information
