# Supplementary material for: Transforming Growth Factor Beta is regulated by a Glucocorticoid-Dependent Mechanism in Denervation Mouse Bone
Source: Sci Rep. 2017 Aug 30;7:9925. doi: 10.1038/s41598-017-09793-y (PMC5577242; doi:10.1038/s41598-017-09793-y)
Supplement: Supplementary file 1 — supplementary information [file 41598_2017_9793_MOESM1_ESM.pdf]

Transforming Growth Factor Beta is regulated by a Glucocorticoid-Dependent Mechanism in Denervation Mouse Bone

Ye Li[1, #], Ligang Jie[2,#], Austin Y Tian[3], Shenrong Zhong[1], Mason Y Tian[4], Yixiu Zhong[1], Yining Wang[1], Hongwei Li[1], Jinlong Li[1*], Xiaoyan Sun[3*] and Hongyan Du[1*]

[1]*School of Laboratory Medicine and Biotechnology, Southern Medical University, Guangzhou, China*

[2]*Department of Chinese Medicine, Guangzhou General Hospital of Guangzhou Command, PLA, Guangzhou, China*

[3]*Department of Neurology, University of Chicago, Chicago, IL, USA*

[4]*College of Dentistry, University of New York, New York, NY, USA*

[#]These authors contributed equally to this work.

Supplementary data

Methods

Micro-CT evaluation for the femurs and lumbar. After been dissected, cleaned, fixed in 10% Millonig's formalin and transferred to100% ethanol, femurs and lumbar which were collected from sham-operated and sciatic nerve crushed mice, were studied using a SkyScan-1176 micro-computed tomography (Bruker Micro-CT, Belgium) system. Scans were performed using PANalytical's Microfocus Tube, with an 8.96-μm voxel size, 45KV, 500 μA and 0.6-degree rotation step (180-degree angular range). For trabecular bone, micro-CT evaluation was performed on a 1-mm region of metaphyseal spongiosa in the distal femur. The regions were located 0.5 mm above the growth plate. Cortical bone measurements were performed on a 1-mm region of the mid-diaphysis of the femur. NR Econ software version 1.6 was used for the 3D reconstruction and viewing of images.

Histochemically testing for ALP and TRACP. The sections were histochemically tested for alkaline phosphatase (ALP) activity using a BCIP/NBT kit (Beyotime Biotechnology, China) and for tartrate-resistant acid phosphatase (TRAP) activity using a TRACP kit (Sigma, USA). The sections were then counterstained with methyl green and mounted in Kaiser's glycerol jelly.

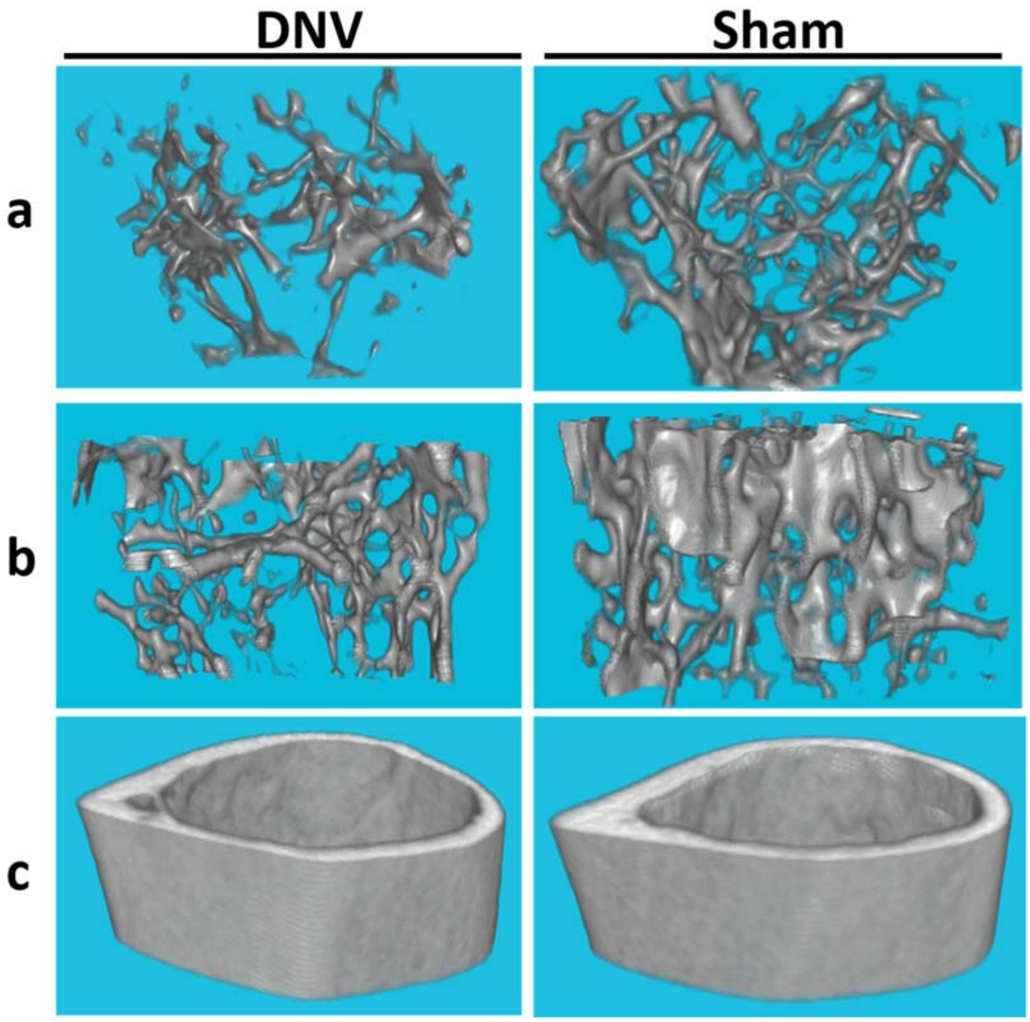

Fig. S-1 Effect of denervation on bone micro-architecture. Micro-CT scan of (a), trabecular bone of femur; (b), trabecular bone of lumbar; (c), cortical bone of femur in sham-operated and sciatic nerve crushed mice.

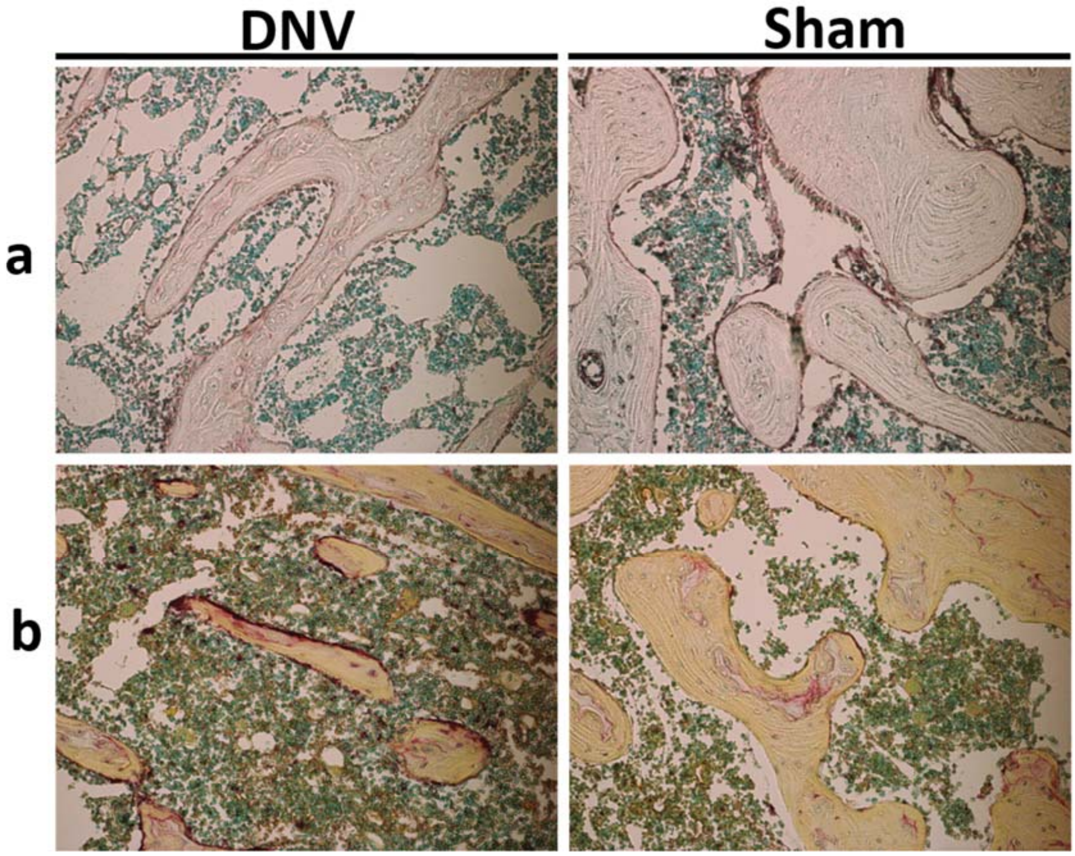

Fig. S-2 Effect of denervation on ALP activity in osteoblast and TRAP activity in osteoclast. (a), ALP activity in sciatic nerve crushed and sham-operated mice; (b), TRAP activity in sciatic nerve crushed and sham-operated mice.

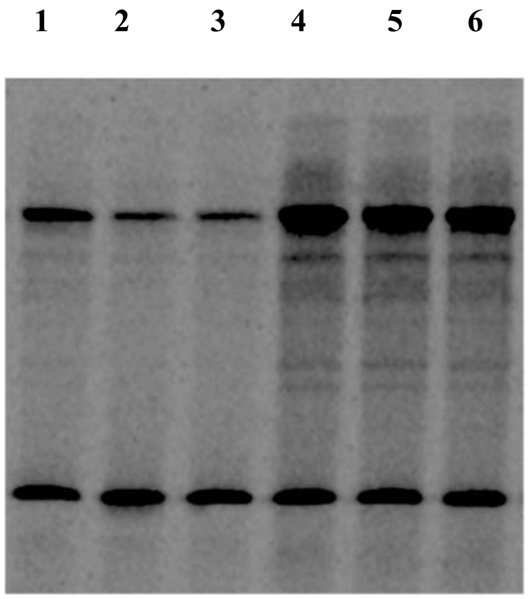

Fig.3a Autoradiography picture for TGF-β1,2,3 and GAPDH. Representative autoradiographic images of the RPA products as resolved on a 6% PAGE sequencing gel. Lane 1, 2 and 3 present sciatic nerve crushed and lane 4, 5 and 6 present sham-operated at 3 weeks in spine, rib and femur respectively.

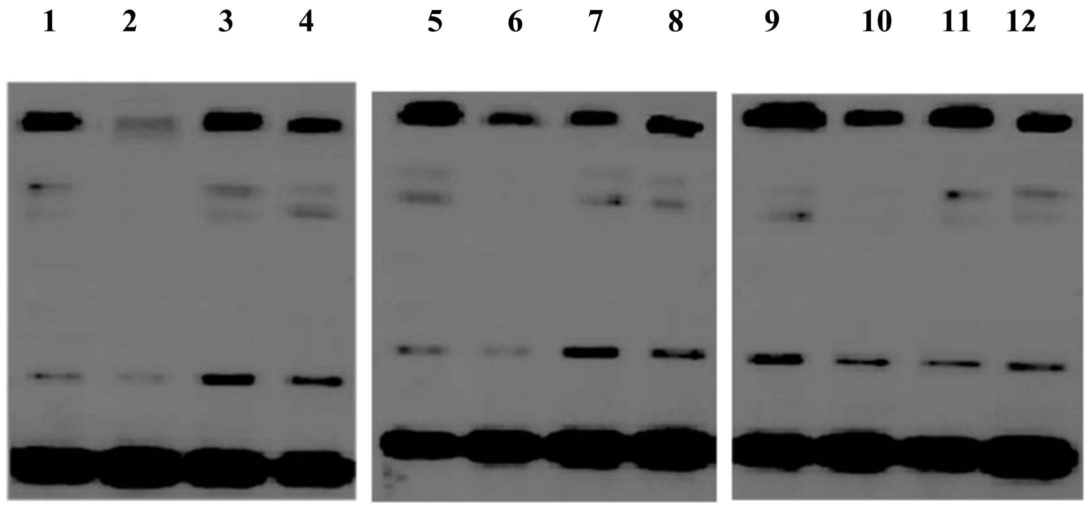

Fig.4c Autoradiography pictures for TGF-β1,2,3 and GAPDH of spine, rib and femur. Representative autoradiographic images of the RPA products as resolved on a 6% PAGE sequencing gel. Lane 1, 5 and 9 present sham; lane 2, 6 and 10 present denervation; lane 3, 7 and 11 present sham with RU 38486 treatment; and lane 4, 8 and 12 present denervation with RU 38486 treatment at 3 weeks in spine, rib and femur respectively.

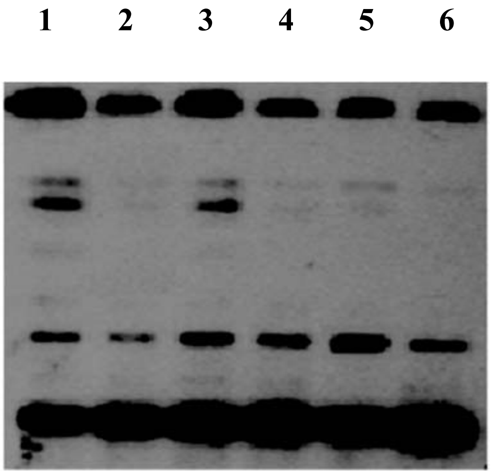

Fig.5b Autoradiography picture for TGF-β1,2,3 and GAPDH of spine, rib and femur. Representative autoradiographic images of the RPA products as resolved on a 6% PAGE sequencing gel. Lane 1, 3 and 5 present Ctrl group, and lane 2, 4 and 6 present DEX-treated group in spine, rib and femur respectively.

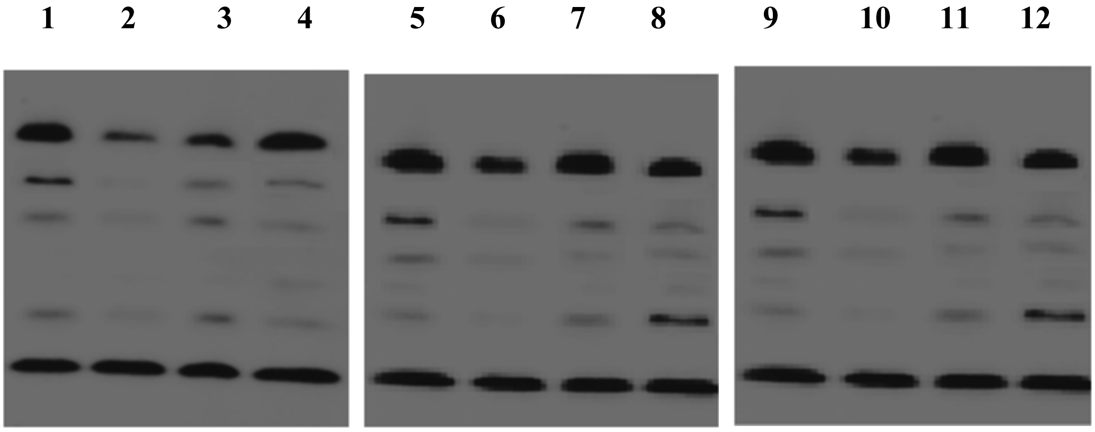

Fig.6b Autoradiography pictures for TGF-β1,2,3 and GAPDH of spine, rib and femur. Representative autoradiographic images of the RPA products as resolved on a 6% PAGE sequencing gel. Lane 1, 5 and 9 present Ctrl; lane 2, 6 and 10 present DEX; lane 3, 7 and 11 present Ctrl with RU 38486 treatment; and lane 4, 8 and 12 present DEX with RU38486 treatment at 3 weeks in spine, rib and femur respectively.
